# Integrative Transcriptomic and Epigenomic Profiling for Signature Identification in Coronary Artery Disease: A Pilot Study

**DOI:** 10.3390/ijms262110437

**Published:** 2025-10-27

**Authors:** Mario Zanfardino, Anna D’Agostino, Ilaria Leone, Katia Pane, Chiara Caselli, Danilo Neglia, Bruna Punzo, Carlo Cavaliere, Andrea Soricelli, Monica Franzese

**Affiliations:** 1 IRCCS SYNLAB SDN, 80143 Naples, Italy; 2 Institute of Clinical Physiology, Department of Biomedical Sciences, Consiglio Nazionale delle Ricerche (CNR), Via G. Moruzzi 1, 56124 Pisa, Italy; 3 Cardiovascular Department, Fondazione Toscana Gabriele Monasterio, Via G. Moruzzi 1, 56124 Pisa, Italy; 4 Department of Medical, Movement and Well-Being Sciences, University of Naples Parthenope, 80133 Naples, Italy

**Keywords:** coronary artery disease, epigenetics, multi-omics, transcriptomics, chromatin accessibility

## Abstract

Coronary Artery Disease (CAD), mainly due to the progressive development of atherosclerotic plaques, is one of the world’s leading causes of mortality and morbidity. A significant percentage of initial events (around 30%) remain fatal to this day despite significant advances in the diagnosis and treatment of cardiovascular diseases (CVDs). Early detection and risk stratification are therefore essential. In this study, we adopted a multi-omics approach integrating transcriptomic (RNA-seq) and epigenomic (ATAC-seq) profiling of peripheral blood mononuclear cells (PBMCs) from a cohort of individuals undergoing clinically indicated cardiac computed tomography angiography (CCTA) to uncover potential novel molecular markers of CAD. We identified 39 genes consistently dysregulated across all CAD subtypes. ATAC-seq analysis revealed distinct chromatin accessibility patterns at CAD-associated loci, with a predominance of quiescent and transcriptionally active states. Validation in an independent cohort confirmed the expression patterns of key Differentially Expressed Genes (DEGs), such as *Claudin 18 (CLDN18)*, supporting the robustness of our findings. Consequently, the integration of multi-omics data allowed us to identify a core gene signature and regulatory patterns associated with disease severity, offering potential biomarkers for clinical risk stratification in patients with CAD.

## 1. Introduction

In industrialised countries, CVDs represent one of the leading causes of death [1,2]. Atherosclerosis represents a key predisposing factor for the development of acute cardiac events. It is characterized by a progressive condition that can result in narrowing (stenosis) or complete obstruction (occlusion) of vessels. One of the most severe consequences of atherosclerosis is CAD, which remains one of the greatest challenges in contemporary cardiovascular medicine [3,4]. Notably, approximately 30% of initial clinical events result in sudden death, which underlines the urgency of early diagnosis and the development of effective prevention strategies [1,5,6]. The ability to accurately predict disease onset, progression, and instability is of paramount importance for risk stratification and early intervention. In this context, a series of studies have highlighted the pivotal role of epigenetic modifications in the pathophysiology of atherosclerosis [7,8,9,10,11]. These studies highlight how epigenetic modifications such as DNA methylation can influence expression patterns that regulate endothelial dysfunction, inflammation, and plaque stability [12,13,14]. Furthermore, epigenetic modifications have also been found in the inflammatory pathway activation, oxidative stress response, and lipid metabolism, all pathways that may play a key role in atherosclerotic plaque formation and progression [15,16,17]. Nevertheless, the identification of reliable epigenetic biomarkers linked to CAD severity and clinical outcomes remains an unmet need. Emerging evidence indicates that specific DNA methylation patterns are associated with disease progression, while histone modifications modulate gene expression in vascular endothelial and smooth muscle cells [18,19]. The integration of high-throughput sequencing with multi-omics data could provide an unprecedented opportunity to characterise the molecular determinants of CAD presence and thus allow the identification of novel precision medicine strategies for targeted intervention [20,21].

This study aimed to investigate genetic and epigenetic factors involved in the pathogenesis of coronary artery disease (CAD) using a multi-omics approach. Specifically, we analyzed RNA-seq and ATAC-seq data from PBMCs to identify differentially expressed genes between healthy individuals and patients with CAD as assessed by CCTA. Additionally, we examined the corresponding chromatin accessibility patterns. The analysis involved an initial cohort of 69 patients stratified into three groups based on CCTA findings: healthy individuals (Control), individuals with non-obstructive coronary artery disease (non_obCAD), and individuals with obstructive coronary artery disease (obCAD). By integrating transcriptomic and epigenomic data, this study seeks to uncover molecular mechanisms underlying atherosclerosis and identify biomarkers for CAD risk stratification. These findings could inform translational applications and the development of diagnostic strategies to prevent cardiovascular disease.

## 2. Results

### 2.1. Clinical Features of the Study Population

Individuals enrolled in the study (*n* = 69) were grouped as controls (CTRL; *n* = 26), obstructive CAD (obCAD; *n* = 15), and non-obstructive CAD (non_obCAD; *n* = 28). For each group, clinical features are reported in Table 1.

### 2.2. Omics Data Characterisation

**Figure 1:**
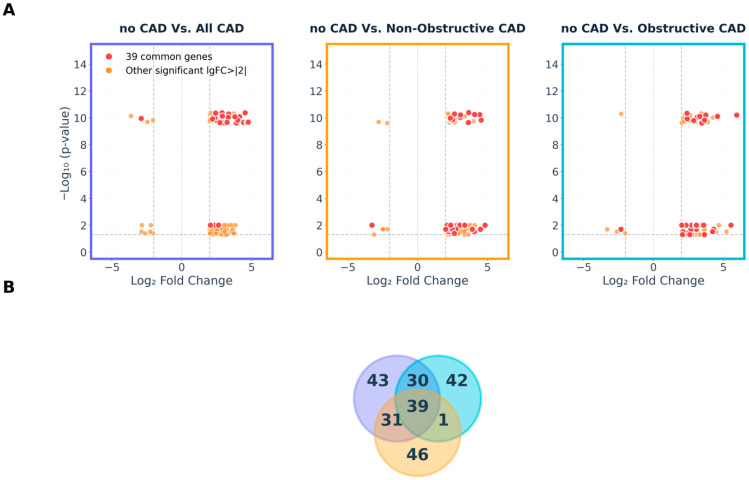
Differential gene expression analysis across CAD patient subgroups and identification of 39 common differentially expressed genes. (**A**) Volcano plots showing DEGs in three comparisons: no CAD vs. All CAD (purple border), no CAD vs. Non-Obstructive CAD (amber border), and no CAD vs. Obstructive CAD (cyan border). Red dots represent the 39 common genes shared across all three comparisons; orange dots indicate other significant genes (|log_2_FC| > 2, *p*-value < 0.05). Dashed lines mark significance thresholds. (**B**) Venn diagram illustrating the overlap of DEGs among the three comparisons, with colour-coded circles matching panel A borders. The central intersection highlights 39 genes consistently dysregulated across all comparisons, representing a core molecular signature. Numbers indicate the count of DEGs unique to or shared between each comparison.

We performed a comprehensive molecular profiling of PBMCs, combining transcriptomic and epigenomic datasets generated through high-throughput sequencing technologies: RNA-seq and ATAC-seq, to identify molecular determinants of CAD (Graphical Abstract). Differential expression analysis was conducted to compare the transcriptomic landscape between each disease group and the controls. A total of significantly (*p* < 0.05) 2245 DEGs were detected when comparing controls versus all CAD cases combined (non-obstructive and obstructive), of which 1425 corresponded to protein-coding genes. A subset of 143 DEGs was identified as highly dysregulated (−2 > logFC > 2) (Supplementary Table S1). When analysing the groups individually, 2125 DEGs (1406 protein-coding) were identified in the control vs. non-obstructive CAD comparison (117 with −2 > logFC > 2, Supplementary Table S2), and 910 DEGs (581 protein-coding) were found in the control vs. obstructive CAD comparison (117 with −2 > logFC > 2, Supplementary Table S3). Additionally, 951 DEGs (664 protein-coding) were observed between non-obstructive and obstructive CAD patients. Notably, 39 genes were found to be commonly dysregulated across all control vs. disease comparisons, suggesting a core molecular signature associated with CAD presence (Figure 1 and Supplementary Table S4).

**Figure 2:**
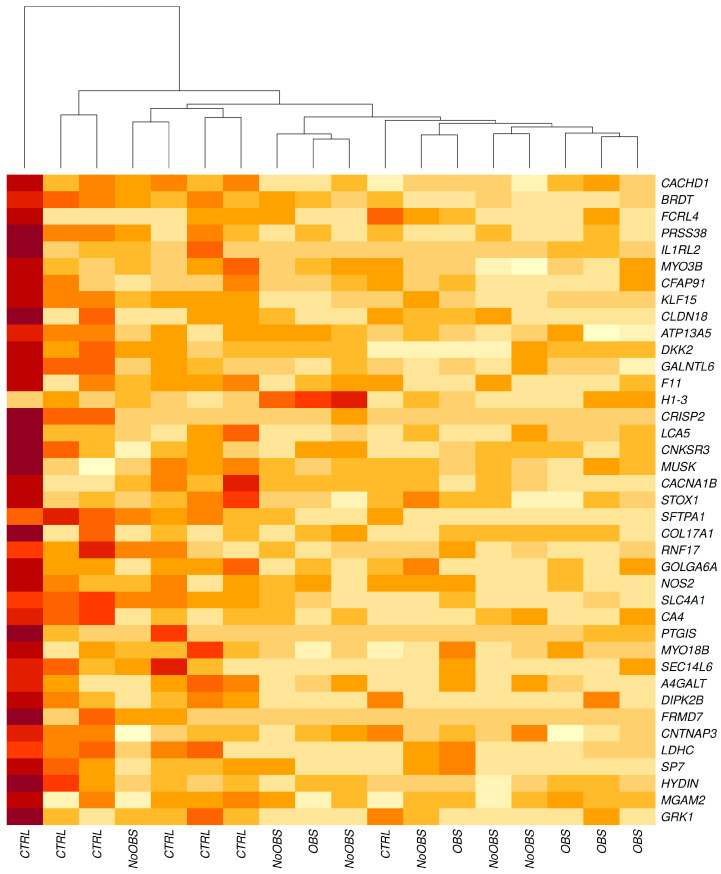
Hierarchical clustering heatmap of differentially expressed genes across experimental conditions. The heatmap displays gene expression profiles for 39 selected genes across multiple sample groups. Samples are grouped into controls (CTRL), Non-Obstructive CAD (NoOBS), and Obstructive CAD (OBS) conditions based on hierarchical clustering analysis. Gene expression levels are represented by color intensity, with darker red indicating higher expression and lighter yellow indicating lower expression. Each row represents an individual gene, and each column represents a sample. The clustering reveals distinct expression patterns between control and experimental conditions, with several genes showing coordinated expression changes. Sample size: CTRL (*n* = 7), NoOBS (*n* = 6), OBS (*n* = 5).

This gene set enabled unsupervised hierarchical clustering that distinctly separated CAD patients from healthy individuals (Figure 2).

The set includes genes such as *CLDN18*, *NOS2*, *SLC4A1*, *CA4*, *COL17A1*, and *SP7*, many of which have been implicated in vascular inflammation, endothelial dysfunction, and extracellular matrix remodelling. Starting from these 39 genes, and following the ChromHMM 18-state model, we selected 5 genes that are associated with high activity regions, actively transcribed regions, genomic regions with regulatory roles, or regions involved in fine-tuned transcriptional changes. *SLC4A1, SFTPA1* fall in regions with strong transcription (5_Tx state) while genes *PRSS38, H1-3,* and *CLDN18* fall instead, respectively, in the states *12_ZNF/Rpts (ZNF genes & repeats)*, *1_TssA (Active TSS),* and *6_TxWk (Weak transcription)*. Epigenomic profiling via integration with chromatin state data from the NIH Roadmap Epigenomics Mapping Consortium revealed diverse chromatin accessibility patterns at loci corresponding to the common DEGs. Among these, 25 genes were mapped to genomic regions exhibiting multiple chromatin states, indicative of regulatory complexity. The most frequently observed chromatin state among these genes was the quiescent/low-activity state (State 18_Quies) (Supplementary Table S5). ATAC-analysis was performed by identifying overlapping genes between differentially accessible regions (Supplementary Table S6) and differentially expressed genes. We focused on the 5 genes selected by integration with the ChromHMM 18-state model, and *CLDN18* emerged as the only one in common with ATAC analysis results.

### 2.3. Omics Data Validation

**Figure 3:**
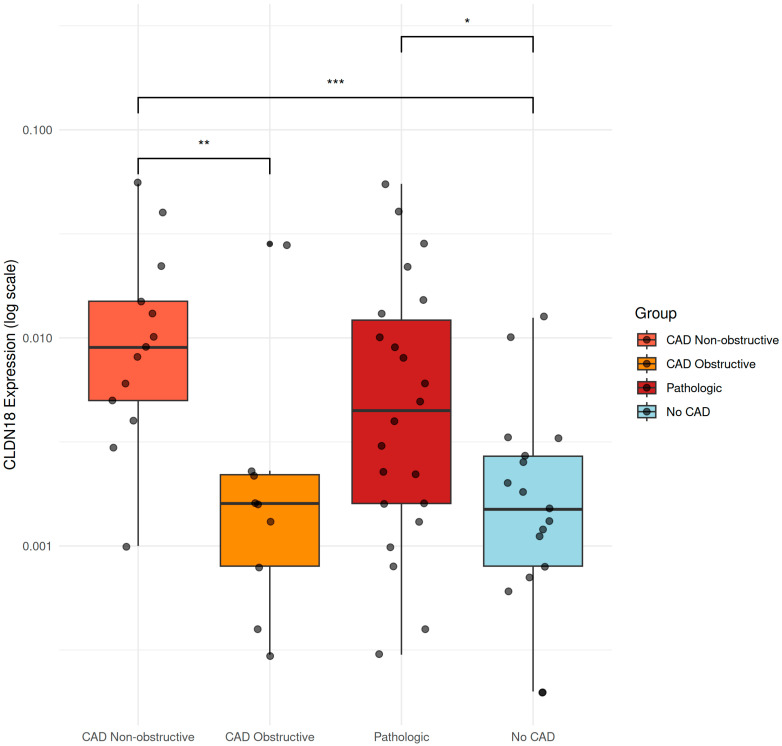
Validation of *CLDN18* gene expression in an independent patient cohort. Box plots showing *CLDN18* expression levels (log scale) across four clinical groups: Non-obstructive CAD, Obstructive CAD, Pathologic (combined CAD groups), and Controls. Individual data points are overlaid on each box plot. Box plots display median (central line), interquartile range (box boundaries), and whiskers extending to 1.5 × IQR. Statistical comparisons between groups are indicated by brackets with significance levels: ** *p* < 0.01, *** *p* < 0.001, * *p* < 0.05. *CLDN18* expression was significantly upregulated in both non-obstructive and pathological patients compared to healthy controls. CAD, coronary artery disease.

The 5 genes obtained from the integration of differentially expressed gene analysis and information from the ChromHMM 18-state model were validated in an independent patient cohort. Specifically, 17 CTRL, 13 non-obCAD, and 10 obCAD subjects were included, and only the *CLDN18* gene resulted significantly differentially expressed (upregulated) in both Non-Obstructive CAD Versus Obstructive CAD and Non-Obstructive CAD Vs. Healthy patients. Moreover, the gene was significantly upregulated in CAD (Non-Obstructive + Obstructive CAD) Versus Healthy patients (Figure 3).

**Figure 4:**
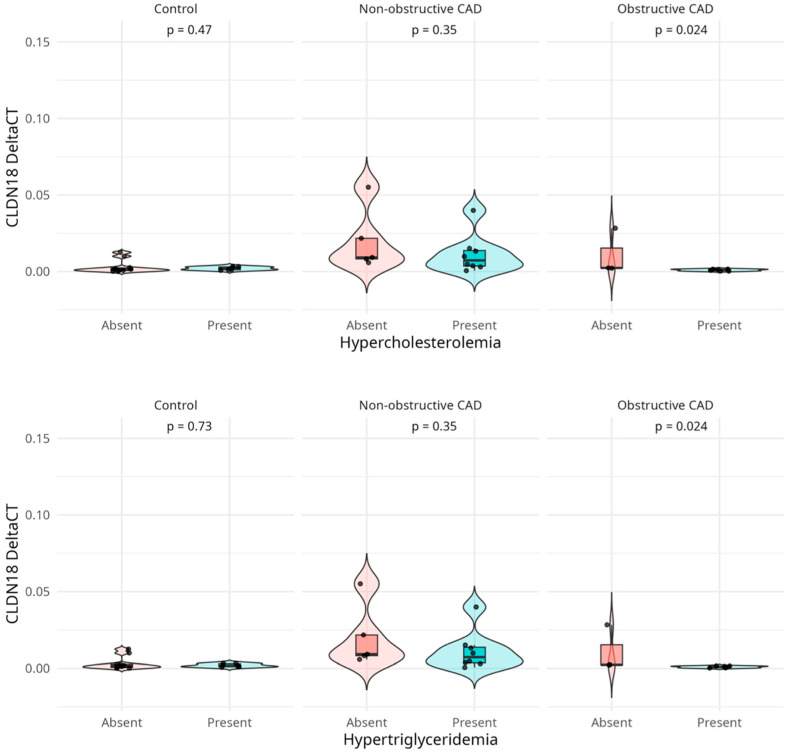
Association between *CLDN18* expression and metabolic conditions in the validated cohort. Violin plots illustrate *CLDN18* ΔCT levels stratified by the presence or absence of hypercholesterolemia (top) and hypertriglyceridemia (bottom) across control subjects, patients with non-obstructive CAD, and patients with obstructive CAD. A significant association (*p* < 0.05) was observed exclusively in the obstructive CAD subgroup, where *CLDN18* expression was higher in the absence of these metabolic risk factors. No significant associations were detected in control subjects or patients with non-obstructive CAD. Note: HTA was defined according to the 2024 ESC Guidelines as office systolic blood pressure ≥ 140 mmHg and/or diastolic blood pressure ≥ 90 mmHg, or current use of antihypertensive medication (Figure 1).

In the validation cohort, *CLDN18* expression was found to be significantly (*p* < 0.05) associated with hypertriglyceridemia and hypercholesterolemia, but this effect was restricted to patients with obstructive CAD. Notably, in vitro validation demonstrated that *CLDN18* expression levels were higher in the absence of these metabolic conditions (Figure 4). No other clinical variables assessed in the descriptive characterisation of the study population showed significant correlations with *CLDN18* expression, underscoring the specificity of this association.

### 2.4. Functional Analysis

Functional enrichment analysis was performed to explore the biological meaning of differentially expressed genes intersected with ATAC-seq-selected genes. Gene Ontology terms do not show significant enrichments, but the *CLDN18* validated gene was found to be involved in the cell–cell interactions pathway, such as the lateral plasma membrane, bicellular tight junction, and apical junction complex (Supplementary Table S7). In the Gene Ontology Biological Process (GOBP) category, we observed a significant enrichment in terms related to regulation of osteoclast development, maintenance of anatomical structure, and tissue homeostasis. These biological processes may reflect systemic alterations associated with chronic inflammation and tissue remodelling, often present in coronary artery disease [22].

Reactome pathway analysis highlighted, for *CLDN18*, categories such as organisation and cell–cell communication, reinforcing the potential role of intercellular connectivity and barrier integrity in CAD pathophysiology, even if these pathways did not reach statistical significance. The KEGG result also showed the underlying Cell adhesion molecule pathway for *CLDN18*. DisGeNET analysis did not return enrichment in cardiovascular-related pathologies, possibly due to the novelty of the identified gene set or the indirect nature of the associated mechanisms. Overall, these findings suggest that the identified genes may be involved in homeostatic processes and intercellular dynamics relevant to CAD progression, although further validation is needed. In particular, under physiological conditions, *CLDN18* contributes to maintaining ion selectivity and epithelial barrier integrity through interactions with scaffolding proteins such as *ZO-1* and occludin. However, aberrant expression of *CLDN18* outside its normal tissue context has been associated with pathological remodelling and inflammation. In cancer models, *CLDN18* dysregulation alters cytoskeletal dynamics and activates signalling cascades involving *RhoA/ROCK* and β-catenin, which modulate cell adhesion, migration, and inflammatory gene expression. By analogy, ectopic expression of *CLDN18* in endothelial cells might perturb junctional organization and promote a pro-inflammatory phenotype, facilitating leukocyte adhesion and transmigration, key early events in atherosclerotic lesion development [23].

## 3. Discussion

Multi-omics integration can represent a powerful approach to unravel the molecular complexity underlying CAD. Integrating multiple layers of information, these approaches not only provide a broader perspective on disease pathophysiology but also enable more precise patient stratification.

Transcriptomic profiling enables the identification of molecular signatures associated with inflammatory processes, oxidative stress, and vascular remodelling. In these pathways, genes such as *TNF-α, IL-6,* and *MMPs* are frequently dysregulated in CAD patients, indicating a systemic inflammatory state and early vascular damage [24]. Complementary epigenomic analyses reveal regulatory changes in genes important for vascular homeostasis and inflammatory responses, which have been linked to arterial stiffness and atherosclerotic plaque formation [25,26,27]. The integration of transcriptomic and epigenomic evidence, therefore, offers not only improved patient stratification but also provides a comprehensive framework for identifying molecular determinants of CAD and potential diagnostic and therapeutic targets. Following this rationale, in our study, we combined RNA-seq and ATAC-seq technologies to explore multi-omics integration, aiming to discover novel biomarkers useful for CAD diagnosis. Transcriptomic analysis revealed a set of differentially expressed genes in patients compared with healthy controls, with enrichment in pathways related to cell–cell interaction and endothelial dysfunction. ATAC-seq analysis, in turn, provided information about changes in chromatin accessibility, revealing regulatory regions possibly involved in gene expression modulation.

The convergence of these omics datasets provides a functional overview of disease-related molecular alterations, strengthening the biological relevance of our findings. Consistent with this approach, recent studies support the value of multi-omics frameworks for discovering new biomarkers and molecular regulators in cardiovascular diseases. For example, Amrute et al. (2024) [28] developed a single-cell variant-to-enhancer-to-gene map for CAD, integrating transcriptomic and microRNA data to identify gene regulatory networks associated with disease-relevant cell types. Their analysis highlighted both well-known transcription factors, such as *FOXC1* and *KLF10*, and novel candidates, including *TBX3* and *TEAD1*, which may contribute to cardiovascular pathophysiology. Although their study did not directly include chromatin accessibility data, the integrative strategy parallels our own approach, in which RNA-seq and ATAC-seq were combined to capture both gene expression changes and regulatory elements potentially controlling these genes. Furthermore, Zhao et al. (2024) [29] employed an integrated multi-omics approach, combining proteomic and single-cell transcriptomic analyses, to investigate the progression of aortic dissection (AD). Their study identified a phenotype-associated macrophage subset that orchestrates the formation of neutrophil extracellular traps (NETs) through the *CXCL3/CXCR2* axis, thereby promoting AD development. Notably, increased NET formation was observed in both the systemic circulation and the aortic microenvironment of AD patients. Inhibition of NET formation ameliorated AD progression and rupture in murine models. These findings underscore the utility of multi-omics profiling in uncovering complex molecular interactions and highlight the potential for therapeutic interventions targeting NETs in vascular diseases.

Thanks to this approach we have identified several candidate genes, and among these genes, *CLDN18* emerged as a particularly interesting candidate. *CLDN18* is generally recognized as a prototypical epithelial tight junction protein and is not typically regarded as a marker of endothelial cells, which line the vascular lumen and play a pivotal role in mediating monocyte adhesion during the early stages of atherogenesis [3,23,30]. However, emerging evidence indicates that endothelial cells can aberrantly express *CLDN18*, particularly the *CLDN18.2* isoform, under pathological conditions [30]. Considering that atherosclerosis is a chronic inflammatory disease, it is conceivable that such a pathological process may promote the induction of *CLDN18* in endothelial cells, thereby not only altering endothelial barrier properties but also influencing the transcriptional programs of circulating PBMCs [31,32].

*CLDN18* was significantly upregulated in CAD patients (in non-obstructive condition) compared with healthy subjects. It showed a differentially accessible chromatin peak [33]. Although not all enriched pathways reached statistical significance, the association with terms such as lateral plasma membrane, bicellular tight junction, and apical junction complex suggests that processes related to intercellular adhesion and endothelial barrier integrity may be disrupted in CAD. Since junctional alterations are well-documented features of cardiovascular diseases, the dysregulation of *CLDN18* could represent a novel contributor to the atherosclerotic process [34,35,36,37,38].

Our observation of *CLDN18* upregulation in CAD patients aligns with studies highlighting endothelial tight junction dysregulation in atherosclerosis. In particular, Nepali et al. (2025) reported that loss of *CLDN18* compromises coronary microvascular barrier integrity, leading to increased permeability, inflammation, and fibrosis [39]. Although *CLDN18* has been less investigated in cardiovascular contexts, recent evidence suggests that its aberrant expression can affect cell–cell adhesion and promote pro-inflammatory signalling [40]. Thus, the upregulation of *CLDN18* in CAD patients observed here may reflect a broader pattern of endothelial remodelling and inflammatory activation, warranting further mechanistic investigation.

## 4. Materials and Methods

### 4.1. Study Population

The study population and recruitment process have been previously described elsewhere [41,42].

Briefly, among patients with suspected or known stable CAD, referred to IRCCS SYNLAB SDN in Naples between July 2018 and March 2022 for clinically indicated CCTA and enrolled in the ‘Studio di biomarcatori in vivo ed in vitro in pazienti affetti da malattie cardiovascolari’ (Observational Study 7/18 OSS SDN), 69 patients were included in the present study. To be eligible for inclusion, participants were required to have a stable sinus rhythm and anatomically and functionally normal heart chambers. Informed consent for participation was obtained from all subjects involved in the study.

### 4.2. Imaging Protocol and Analysis

As already described [43], all patients underwent CCTA using a third-generation dual-source multidetector CT scanner (Somatom Force, Siemens Healthineers, Forchheim, Germany). The system offers an effective temporal resolution of 66 ms per reconstructed axial slice, enabling the acquisition of high-quality, motion-free datasets in both diastolic and systolic phases, regardless of heart rate. Initially, a non-contrast-enhanced high-pitch spiral scan (FLASH mode) was performed to assess coronary artery calcium (CAC) using the following parameters: 3 mm slice thickness, 3 mm increment, and a small cardiac field of view (FOV). Subsequently, contrast-enhanced CCTA was performed using intravenous administration of 50 mL of iodinated contrast agent (Iomeprol 400 mg/mL, Iomeron 400, Bracco, Milan, Italy) at a flow rate of 5 mL/s, followed by a 50 mL saline flush at the same rate. Scans were acquired with retrospective ECG gating and prospective ECG-based tube current modulation (targeting the 25–75% window of the RR interval). To optimize image quality while minimizing radiation exposure, automated attenuation-based tube current modulation (CARE Dose4D) and tube voltage selection (CARE kV) were applied. Post-processing was performed using “Syngo. Via” software (version VB60A_HF04, Siemens Healthineers) to calculate the Agatston calcium score and to assess coronary stenosis severity for patient stratification.

Based on the outcome of the CCTA investigation, the study population (*n* = 69) was stratified into three groups: patients without any detectable coronary plaque were classified as Control (Control *n* = 26), obstructive CAD (≥50% stenosis in at least one major coronary vessel; *n* = 15), non-obstructive CAD (<50%; *n* = 28).

### 4.3. PBMC Isolation

Peripheral blood (PB) samples for experimental purposes were obtained and collected in 3 mL EDTA vacutainer tubes (Becton Dickinson, Franklin Lakes, NJ, USA, Catalog. #367835). White blood cells (WBC) were derived by PB dilution 1:20 with VersaLyse Solution (A09777, Beckman-Coulter, Brea, CA, USA) for red blood cell removal by osmotic shock. PBMCs were obtained by centrifugation on density gradient media (Pancoll^®^ density 1077 g/L, PanBiotech, Aidenbach, Germany) at 400× *g* for 30 min. The biological samples included in this study were provided and processed by the Biobank of the SDN institute [44,45].

### 4.4. RNA Isolation

RNA from PBMCs of recruited patients were isolated using Trizol Reagent protocol (Thermo Fischer Scientific, Waltham, MA, USA). The total RNA extracted was evaluated using QubitTM 4 Fluorometer (Thermo Fischer Scientific).

### 4.5. cDNA Synthesis and Real-Time Quantitative PCR

cDNA syntheses were performed using SuperScriptTM III First–Strand Synthesis SuperMix kit (Thermo Fisher Scientific) according to the manufacturer’s instructions. The RPS18 gene was used as a housekeeping gene. RT-qPCR experiments were performed using C1000 Touch Thermal Cycler (Bio-Rad, Hercules, CA, USA) using iQ SYBR Green Supermix (#1708882, Bio-Rad, Hercules, CA, USA). The following thermal protocol has been applied: initial denaturation (95 °C, 3 min), 40 cycles of denaturation (95 °C, 10 s), annealing (60 °C, 30 s) and elongation (72 °C, 30 s), final elongation (72 °C, 10 min) and a final hold (4 °C). The melting curve was generated in the range of 60–95 °C. The reaction volume was 10 µL. Each reaction was performed in duplicate. Samples were normalized to their RPS18 level using the 2^−ΔCt^ method. Two independent experiments were performed for each RT–qPCR. Data were analyzed using Biorad CFX Maestro version 1.0 (Bio-Rad, Hercules, CA, USA). Oligonucleotides used for RT-qPCR are shown in Supplementary Table S8.

### 4.6. Fast-ATAC Sequencing

As for the Fast-ATAC sequencing protocol, the procedure described in Corces et al. 2016 [46] was used. In brief, 5000 sorted cells in FACS Buffer of 11 patients were pelleted by centrifugation at 500 RCF for 5 min at 4C in a pre-cooled fixed-angle centrifuge. All supernatant was removed using two pipetting steps, being careful not to disturb the invisible cell pellet. 50 μL transposase mixture (25 μL of 2x TD buffer, 2.5 μL of TDE1, 0.5 μL of 1% digitonin, 22 μL of nuclease-free water) (Cat# FC-121-1030, Illumina, San Diego, CA, USA; Cat# G9441, Promega, Madison, WI, USA) was added to the cells, and the pellet was disrupted by pipetting. Transposition reactions were incubated at 37 °C for 30 min in an Eppendorf ThermoMixer with agitation at 300 RPM. Transposed DNA was purified using a QIAgen MinElute Reaction Cleanup kit (Cat# 28004, Hilden, Germany), and purified DNA was eluted in 10 μL elution buffer (10 mM Tris-HCl, pH8). Transposed fragments were amplified and purified as described previously [47] with modified primers [48]. Library quantification was performed using qPCR before sequencing. All Fast-ATAC libraries were sequenced using paired-end, dual-index sequencing on a NextSeq with 76 × 8 × 8 × 76 cycle reads.

### 4.7. RNA-Seq Analysis and Chromatin State Analysis

A total of 18 samples were analyzed, including 7 controls, 6 non-obstructive, and 5 obstructive samples. Adapter sequences and low-quality reads were removed from the raw FASTQ files before alignment. Filtered reads were aligned to the GRCh38 reference genome using Bowtie2 (v2.5.3) [49], achieving an overall alignment rate of 85–90%. SAM files were converted to BAM format using Samtools (v1.19.2). Gene-level quantification was performed with featureCounts from the Rsubread (v2.10.4) R package, using the hg38 NCBI RefSeq GTF annotation. Only uniquely mapped reads were retained, and features with zero counts across all samples were discarded. A range of 79–81% of reads were successfully assigned to features. Read count normalization was performed using estimateSizeFactors in the DESeq2 (v1.36.0) R package [50]. Differential expression analysis (DEA) was conducted in DESeq2 by comparing the following conditions: Controls Vs. Non-Obstructive + Obstructive, Controls Vs. Non-Obstructive and Controls vs. Obstructive. A threshold of *p* < 0.05 was applied to identify DEGs. Genes with a log-fold change (logFC) outside the range of −2 > logFC > 2 were considered highly differentially expressed. The chromatin state of DEGs was assessed using data from the NIH Roadmap Epigenomics Mapping Consortium (https://egg2.wustl.edu/roadmap/web_portal/, 3 February 2025). Hierarchical clustering was performed using the complete linkage method in the sats R package (v4.3.1). Genes were classified into multiple chromatin states, with particular attention to Quiescent/Low (18_Quies) and Weak Repressed PolyComb (17_ReprPCWk) states. All data analyses and graphical representations were conducted in R (v4.2.1). Hierarchical clustering and heatmaps were generated for genes identified as differentially expressed across all comparisons. A Venn diagram was used to determine overlapping DEGs across conditions.

### 4.8. ATAC-Seq Analysis

Raw ATAC-seq paired-end reads of a total of 11 patients were aligned to the GRCh38 reference genome (without alternative contigs) using Bowtie2 with default parameters. The resulting SAM files were converted to BAM format using SAMtools. Quality control and filtering steps were performed sequentially on the aligned reads. PCR duplicates were removed using SAMtools rmdup. Low-quality mappings were filtered out by removing reads with mapping quality scores below 30 (MAPQ < 30). Mitochondrial reads were excluded, and regions overlapping with the ENCODE unified blacklist for GRCh38 were removed using “BEDTools intersect”. Peak calling was performed using MACS2 with the following parameters: “–nomodel” flag for ATAC-seq data, -f BAMPE for paired-end reads, and a *p*-value threshold of 1 × 10^−5^. A consensus peak set was generated by concatenating all individual peak files, sorting by genomic coordinates, and merging overlapping peaks using “BEDTools merge”. To quantify accessibility at consensus peaks, featureCounts was used to count reads in each peak region across all samples. Differential accessibility analysis was performed using DESeq2 with default parameters, comparing Controls and Non-Obstructive conditions. Peaks with adjusted *p*-value < 0.05 were considered significantly different between conditions. Peak annotation was performed using ChIPseeker with UCSC hg38 known genes as a reference. Integration with RNA-seq data was performed by identifying overlapping genes between differentially accessible regions and differentially expressed genes.

The resulting gene sets were analyzed for enrichment of Gene Ontology terms, KEGG pathways, and Reactome pathways using clusterProfiler. Disease ontology enrichment was performed using the DOSE R package (v4.2).

### 4.9. Statistical Analysis

Descriptive statistics were used to summarise the characteristics of the study population across the three diagnostic groups (Control, Non-obstructive CAD, and Obstructive CAD). Continuous variables were reported as mean ± standard deviation and categorical variables as counts and percentages. Statistical analyses were performed to assess the association between *CLDN18* expression levels and clinical variables. For continuous variables, group comparisons were conducted using one-way ANOVA or the Kruskal-Wallis test, depending on data distribution, while correlations with *CLDN18* expression were evaluated using Pearson or Spearman correlation coefficients, as appropriate (correlation analyses were conducted separately within each diagnostic group). For categorical variables, comparisons between groups were performed using the chi-square test, Fisher’s exact test, or the Wilcoxon rank-sum test, as applicable. *p*-values < 0.05 were considered statistically significant.

## 5. Conclusions

The absence of cardiovascular disease enrichment for this gene in DisGeNET may reflect the limited characterization of certain genes or regulatory elements in curated databases, rather than a lack of biological relevance. Additional limitations include the need for larger cohorts and independent replication to confirm the robustness and generalizability of our findings. Crucially, functional validation in vitro and in vivo will be required to elucidate the mechanistic role of *CLDN18* and related pathways in non-obstructive CAD.

Despite these limitations, our results support the value of integrating transcriptomic and epigenomic data to capture early and potentially indirect mechanisms underlying CAD. This integrative strategy yields a more dynamic portrait of disease-associated molecular alterations, facilitating the identification of novel biomarkers for diagnosis and risk stratification. Future research should focus on validating the identified biomarkers in larger, independent cohorts and exploring their mechanistic role in CAD pathogenesis. Integrating multi-omics data with longitudinal clinical information could also facilitate the development of predictive models to personalize therapeutic strategies for cardiovascular risk management.

## Figures and Tables

**Table 1:** Baseline Characteristics of Study Population.

	Control (*n* = 26)	Non-Obstructive CAD (*n* = 28)	Obstructive CAD (*n* = 15)	*p*-Value
Demographics				
Age (years)	59.8 (±10.5)	63.5 (±8.6)	65.3 (±12.8)	NS
Male sex, *n* (%)	11 (42.3%)	19 (67.9%)	13 (86.7%)	<0.05
Weight (kg)	72.2 (±15.0)	79.5 (±15.0)	84.2 (±14.8)	<0.05
Height (m)	1.7 (±0.1)	1.7 (±0.1)	1.7 (±0.1)	NS
BMI (kg/m^2^)	25.6 (±3.8)	27.2 (±4.9)	28.5 (±3.6)	NS
BSA (m^2^)	1.8 (±0.2)	1.9 (±0.2)	2.0 (±0.2)	NS
Cardiovascular Risk Factors				
Family history, *n* (%)	17 (65.4%)	13 (46.4%)	12 (80.0%)	NS
Current smoking, *n* (%)	2 (7.7%)	8 (28.6%)	6 (40.0%)	<0.05
Diabetes mellitus, *n* (%)	0 (0.0%)	4 (14.3%)	1 (6.7%)	NS
Hypertension, *n* (%)	18 (69.2%)	21 (75.0%)	15 (100.0%)	NS
Hypercholesterolemia, *n* (%)	13 (50.0%)	17 (60.7%)	12 (80.0%)	NS
Hypertriglyceridemia, *n* (%)	12 (46.2%)	18 (64.3%)	12 (80.0%)	NS
Obesity, *n* (%)	8 (30.8%)	5 (17.9%)	7 (46.7%)	NS
Clinical Parameters				
Systolic BP (mmHg)	127.2 (±13.4)	129.9 (±11.1)	136.2 (±21.4)	NS
Diastolic BP (mmHg)	79.9 (±9.5)	80.4 (±8.8)	82.0 (±8.9)	NS
Creatinine (mg/dL)	0.9 (±0.2)	0.9 (±0.2)	1.0 (±0.2)	<0.05
Medications				
Statins, *n* (%)	8 (30.8%)	13 (46.4%)	9 (60.0%)	NS
Other Variables				
Menopause, *n* (%)	2 (7.6%)	4 (14.2%)	1 (6.7%)	NS
COVID-19 history, *n* (%)	18 (69.2%)	22 (78.5%)	11 (73.3%)	NS
COVID-19 vaccination, *n* (%)	22 (84.6%)	27 (96.4%)	12 (80%)	NS

## Data Availability

Data is contained within the article and Supplementary Materials and at https://www.ncbi.nlm.nih.gov/sra/?term=PRJNA1330009 (accessed on 10 September 2025).

## Supplementary Materials

The following supporting information can be downloaded at: https://www.mdpi.com/article/10.3390/ijms262110437/s1.
